# Diabetic Dyslipidemia and Its Determinants Among People With Diabetes in South Africa: Protocol for a Systematic Review and Meta-Analysis

**DOI:** 10.2196/82716

**Published:** 2026-02-19

**Authors:** Mashudu Nemukula, Fentahun Adane, Arun Kumar Malaisamy, Neel Sarovar Bhavesh, Olebogeng Harold Majane, Sechene Stanley Gololo

**Affiliations:** 1 Department of Biochemistry and Biotechnology, School of Science and Technology, Sefako Makgatho Health Sciences University, 51 Molotlegi Street, Pretoria, 0204, South Africa, 27 0813505474; 2 Transcription Regulation Group, Integrative Biology, International Centre for Genetic Engineering and Biotechnology, New Delhi, India; 3 Department of Biomedical Sciences, School of Medicine, Debre Markos University, Debre Markos, Ethiopia; 4 Department of Human Physiology, School of Medicine, Sefako Makgatho Health Scienes University, Pretoria, South Africa

**Keywords:** diabetic dyslipidemia, systematic review, meta-analysis, diabetes, PRISMA

## Abstract

**Background:**

Diabetic dyslipidemia (DD), characterized by a classical triad of abnormal lipid profiles among the diabetic population, presents a major public health concern in South Africa, particularly among Black South Africans. The increasing prevalence of DD significantly contributes to the development of atherosclerotic cardiovascular disease. With the incidence of diabetes rising from 4.5% in 2010 to 12.7% in 2021, urgent preventive measures and effective treatments are crucial to tackle the risk of premature mortality.

**Objective:**

This systematic review and meta-analysis protocol aims to examine the existing literature on DD, providing an understanding of its prevalence and associated predictors among the diabetic population in South Africa, with the intention of informing more effective clinical and public health interventions.

**Methods:**

The protocol is registered in PROSPERO (International Prospective Register of Systematic Reviews) and will adhere to the PRISMA (Preferred Reporting Items for Systematic Reviews and Meta-Analyses) guidelines. The available literature on DD will be systematically searched in common scholarly databases and reviewed accordingly. All published and unpublished studies conducted in South Africa prior to 2024 and written in English will be included. Two members (MN and FA) of the review team will independently screen the studies identified through the database search and assess risk of bias using the revised JBI critical appraisal tools. The review will integrate both quantitative and qualitative data synthesis. Results from both qualitative and quantitative data synthesis will be presented through forest plots, subgroup forest plots, and summary tables, which will present findings on pooled prevalence, odds ratios for predictors, heterogeneity statistics, and sensitivity analyses.

**Results:**

The protocol was finalized in January 2025. The literature search was conducted between October 2024 and March 2025. Title and abstract screening began in April 2025, and full-text review was completed by July 2025, with data extraction scheduled for completion by September 2025. The completion of statistical analyses is expected by October 2025. We anticipate submission of the completed systematic review and meta-analysis for publication in December 2025.

**Conclusions:**

The findings of the study protocol will inform the design of targeted interventions and policies aimed at advancing the management of DD and subsequently reducing the increased risk of atherosclerotic cardiovascular disease among the diabetic population.

## Introduction

Diabetic dyslipidemia (DD) is one of the major public health concerns, particularly in patients with diabetes. It is an established determinant for the development of atherosclerotic cardiovascular disease (ACVD) and contributes to increased rates of morbidity and mortality. Despite international efforts to improve the management of diabetes, DD remains a significant contributor to the burden of cardiovascular morbidity, especially in developing countries [1,2].

The prevalence of DD is high in developing countries, where the burden of diabetes is on the rise [3]. In South Africa, DD is a growing concern, with several studies indicating that a significant proportion of individuals with diabetes have lipid abnormalities, namely hypertriglyceridemia, decreased levels of high-density lipoprotein cholesterol (HDL-C), and elevated levels of low-density lipoprotein cholesterol (LDL-C). This lipid profile aggravates the risk of developing ACVD, which is emerging as the leading cause of preventable mortality in the country [4-6].

In South Africa, diabetes and its associated metabolic disorders, including lipid abnormalities, are increasingly prevalent among certain ethnic groups, with a notably higher prevalence seen among Black South Africans [7,8]. According to the International Diabetes Federation, the prevalence of diabetes in South Africa has notably increased from 4.5% in 2010 to 12.7% in 2019, with an estimated 4.58 million people aged 20‐70 years affected, of whom 52.4% are undiagnosed [9,10]. Moreover, South Africa has the second-highest number of people living with type 2 diabetes mellitus in sub-Saharan Africa [11]. These metabolic disorders are significant determinants of the national burden of ACVD and premature mortality, with diabetes-related cardiovascular death predicted to increase significantly in the coming years if preventive measures and effective treatment strategies are not implemented [12-14].

Despite the ongoing public health initiatives and policies aimed at addressing diabetes and the determinants of cardiovascular disease, the prevalence of DD continues to rise, particularly among young individuals and those with poor glycemic control [15,16]. Existing studies have shown that DD is prevalent among diabetic patients who have suboptimal glycemic control and engage in unhealthy lifestyle choices, such as poor dietary habits and sedentary lifestyles [17,18]. Therefore, there is a need for updated studies to better understand the epidemiology of DD, particularly in the context of South Africa, to inform targeted intervention strategies and improve patient outcomes.

Predictors of DD include sociodemographic factors such as age, gender, ethnicity, education level, and household income, as well as the presence of other comorbid conditions, including hypertension, obesity, and chronic kidney disease [19,20]. Moreover, psychological factors such as stress, as well as sedentary lifestyle factors, contribute to the development and progression of DD among individuals with diabetes mellitus [21]. Understanding these predictors is important for developing more effective interventions to manage lipid abnormalities in diabetes mellitus.

The aim of this systematic review and meta-analysis is to estimate the pooled prevalence of DD among the diabetic population of South Africa and to assess the association between DD and its predictors. The review intends to provide an understanding of how certain predictors influence lipid profiles in diabetes mellitus. In addition, the review also seeks to inform targeted interventions aimed at reducing the incidence of DD and its associated cardiovascular risks in South Africa.

## Methods

### Overall Approach

The systematic review will be conducted following the PRISMA (Preferred Reporting Items for Systematic Reviews and Meta-Analyses) guidelines [22] on studies reporting on DD among people with diabetes in South Africa. Following this approach will ensure a transparent and systematic process for identifying, selecting, and including relevant studies. The protocol is registered with PROSPERO (International Prospective Register of Systematic Reviews; registration number CRD42024614221).

### Population of Interest

The population of interest will include South Africans diagnosed with diabetes mellitus, including both type 1 and type 2 diabetes. The emphasis is on the diabetic population presenting with DD, characterized by lipid abnormalities such as hypertriglyceridemia, reduced HDL-C, and elevated LDL-C. Nondiabetic individuals will not be included in the review.

### Eligibility Criteria

Studies reporting data on DD (hypertriglyceridemia, low HDL-C, and elevated LDL-C) among South Africans with diabetes mellitus will be considered. Observational studies such as cross-sectional, cohort, and case-control studies will be included. Studies reporting data on the prevalence or predictors of diabetic dyslipidemia will be included. Studies conducted among non-South Africans, including those involving nondiabetic populations, will be excluded. Reviews, case reports, editorials, and conference abstracts reporting on diabetic dyslipidemia will be disregarded. Studies without relevant lipid profile data will also be excluded.

### Search Strategy

Studies for this systematic review and meta-analysis will be accessed through electronic and other relevant sources. We intend to use common database searches, such as PubMed/MEDLINE, Embase, Scopus, Web of Science, ScienceDirect, African Index Medicus, and Google Scholar, as well as local databases like African Journals Online and SABINET, to identify relevant articles. In addition, we will search publicly accessible institutional repositories such as open-access libraries from the university community, which include publicly available theses and dissertations, for identification of eligible studies. Restricted-access repositories and private databases will not be used for identifying eligible studies. Only publicly accessible sources will be consulted, ensuring full compliance with institutional privacy and data protection. Our search will include all published and unpublished studies conducted in South Africa before December 2024 and written in the English language. The searches will be conducted using Medical Subject Headings (MeSH) terms, combined key terms, text words, and search strings taken from the review questions. To access the records, the following combination key terms will be used: (“Diabetic dyslipidemia” AND “lipid profile” OR “HDL-C” OR “LDL-C” OR “Triglycerides”) AND (“Diabetes mellitus” OR “T1DM” OR “T2DM”) AND (“predictors” OR “risk factors” OR “determinants” OR “contributing factors”) AND (“South Africa”). After identifying the key relevant studies, their references will also be searched (backward reference searching). Similarly, other studies that cited them will be retrieved (forward reference searching).

### Article Screening and Data Extraction

Two members of the review team (MN and FA) will independently screen the titles and abstracts of all studies identified through the database search. Prior to screening, all identified citations will be exported to Microsoft Excel (Microsoft Corp) for duplicate identification and removal. Duplicate records will be identified using sorting functions, conditional formatting, and unique reference identifiers to highlight repeated entries. A manual verification step will be performed to ensure complete and accurate removal of duplicates before title and abstract screening. Prior to the initiation of full screening, MN and FA, the two reviewers, will conduct a pilot assessment phase using a random sample of approximately 10%‐20% of the retrieved studies to enhance and harmonize the application of the eligibility criteria. Interrater agreement will be evaluated using the Cohen κ statistic, and a threshold of κ≥0.70 will be considered acceptable for proceeding with full screening. Any disagreements arising during the pilot phase will be reviewed and discussed between the two reviewers to ensure a consistent interpretation of the criteria. Following the pilot assessment phase, a full-text review will be conducted for studies that appear to meet the inclusion criteria based on their title and abstract. Full-text articles will then be evaluated to determine whether they meet the predefined inclusion and exclusion criteria. Articles reporting on the South African population with diabetes mellitus and DD markers and outcomes will be included. Studies not involving South African populations, as well as nondiabetic populations or those with gestational diabetes or prediabetes, will be excluded. Nonoriginal research and studies that lack adequate data on lipid profiles or cardiovascular disease outcomes will be excluded. All essential data will be extracted and recorded in Microsoft Excel using a checklist data extraction format made by the review team. The same members of the review team will independently extract the data using a standardized form. Study characteristics, population characteristics, exposure and intervention, outcomes, and effect estimates data will be collected and recorded. The third reviewer, AKM, will adjudicate any disagreements between the two reviewers and provide the final decision if consensus cannot be reached. Additionally, any discrepancies will be reviewed and discussed with the third reviewer. All data will be securely stored, with regular backups in place to safeguard against any potential loss. In cases where critical data are missing, the corresponding authors of the studies will be contacted for clarification or additional information. If the missing data cannot be retrieved, the impact of these gaps in the review will be discussed, and sensitivity analyses will be performed to assess the robustness of our review findings.

### Risk of Bias Assessment

The review will incorporate a rigorous assessment of risk of bias to evaluate the quality of the selected studies, using tailored tools that depend on the study design. For observational studies such as case-control, cross-sectional, and cohort studies, the Newcastle-Ottawa Scale will be used to evaluate bias in terms of selection methods, comparability, and outcomes [23]. Studies will be classified as high, moderate, or low quality. In the case of randomized controlled trials, the Cochrane Risk of Bias Tool will assess the bias, rating them as low, high, or unclear risk. In addition, an adapted JBI checklist will be used to assess cross-sectional studies, focusing on inclusion criteria, exposure and outcome measurement reliability, and statistical appropriateness [24]. Two independent reviewers will evaluate each study to determine and resolve any discrepancies, with a pilot assessment ensuring consistency. High-risk studies will be disqualified from quantitative data synthesis and will either be included in the qualitative data synthesis or form part of the sensitivity analysis. The overall risk of bias results will be summarized in a table format, and the findings will be interpreted considering the quality of the study and the level of bias.

### Data Synthesis and Statistical Analysis

The review will integrate both quantitative and qualitative data synthesis, depending on the availability of data and consistency across studies. For qualitative data synthesis, the descriptive synthesis method will be used to provide a detailed overview of the included studies, highlighting key aspects such as study design, population demographics, sample sizes, and regional distribution across South Africa. A table will be developed, noting the author, year, study location, type of diabetes mellitus, prevalence rates of DD, and reported predictors. Trends and patterns, including regional prevalences, will also be documented. A specified set of predictors of DD will be extracted from all the eligible studies. To avoid selection bias and ensure consistent data extraction, predictors will be categorized into the following groups: sociodemographic factors (age, gender, level of education, and socioeconomic status), lifestyle factors (smoking, alcohol consumption, physical inactivity), clinical and cardiometabolic factors (BMI, blood pressure, hypertension, obesity, duration of diabetes, and glycemic control), and laboratory markers (glycated hemoglobin, total cholesterol, triglycerides, HDL-C, and LDL-C). A quantitative meta-analysis of predictors will be carried out if a minimum of 3 studies report the same predictors with comparable definitions and effect measures; otherwise, they will be reported qualitatively. For quantitative data synthesis, a meta-analysis will be conducted to estimate the overall prevalence of DD and assess the strength of relationships between DD and predictors among South Africans with diabetes mellitus. A random effects model will be used due to the expected heterogeneity in characteristics of the population, including the definition of dyslipidemia across studies. The pooled prevalence will be calculated, and a meta-analysis of logistic regression will be conducted to assess predictors. Heterogeneity will be examined using the Cochran Q test and *I*² statistics, along with subgroup and sensitivity analyses, to identify potential causes of variance. For articles that cannot be meta-analyzed, qualitative data synthesis will be used to identify trends and variations across populations and outcomes. Publication bias will be assessed using funnel plots (with at least 10 papers included) and Egger or Begg tests. The analysis will be conducted using the R (version 4.3.0 or higher) package for flexibility in handling effect sizes, heterogeneity analysis, and visualization. Stata (version 18) will also be used for meta-regression. Power analysis will also be conducted to ensure a sufficient sample size for detecting statistically significant effects. Receiver operating characteristic curve analysis will be conducted to evaluate the diagnostic accuracy of specific predictors and predictive models [25,26]. Results from both qualitative and quantitative data synthesis will be presented through forest plots, subgroup forest plots, and summary tables, presenting findings on pooled prevalence, odds ratios for predictors, heterogeneity statistics, and sensitivity analyses [27-30].

### Subgroup and Sensitivity Analysis

To minimize random discrepancies, a pilot data extraction will be conducted on subgroups and subsets of studies to ensure consistency and accuracy in the data extraction process. This will be done based on the areas in which the studies were conducted. In addition, all the extracted data will be safely deposited, and all files will be backed up to prevent any data loss.

### Ethical Considerations

The study is a systematic review that does not involve direct interaction with human participants or the use of identifiable personal data; therefore, ethical approval will not be required.

### Dissemination

The review findings will be submitted for publication in a peer-reviewed scientific journal specializing in diabetes, cardiovascular health, or public health. The communication plan details include preparing a manuscript for submission to a reputable journal, presenting the review findings at conferences, and engaging with institutions and the community.

## Results

The protocol was finalized in January 2025. The literature search was conducted between October 2024 and March 2025. Title and abstract screening began in April 2025, and full-text review was completed by July 2025, with data extraction scheduled for completion by September 2025. The completion of statistical analyses is expected by October 2025. We anticipate submission of the completed systematic review and meta-analysis for publication in December 2025.

**Figure 1.**
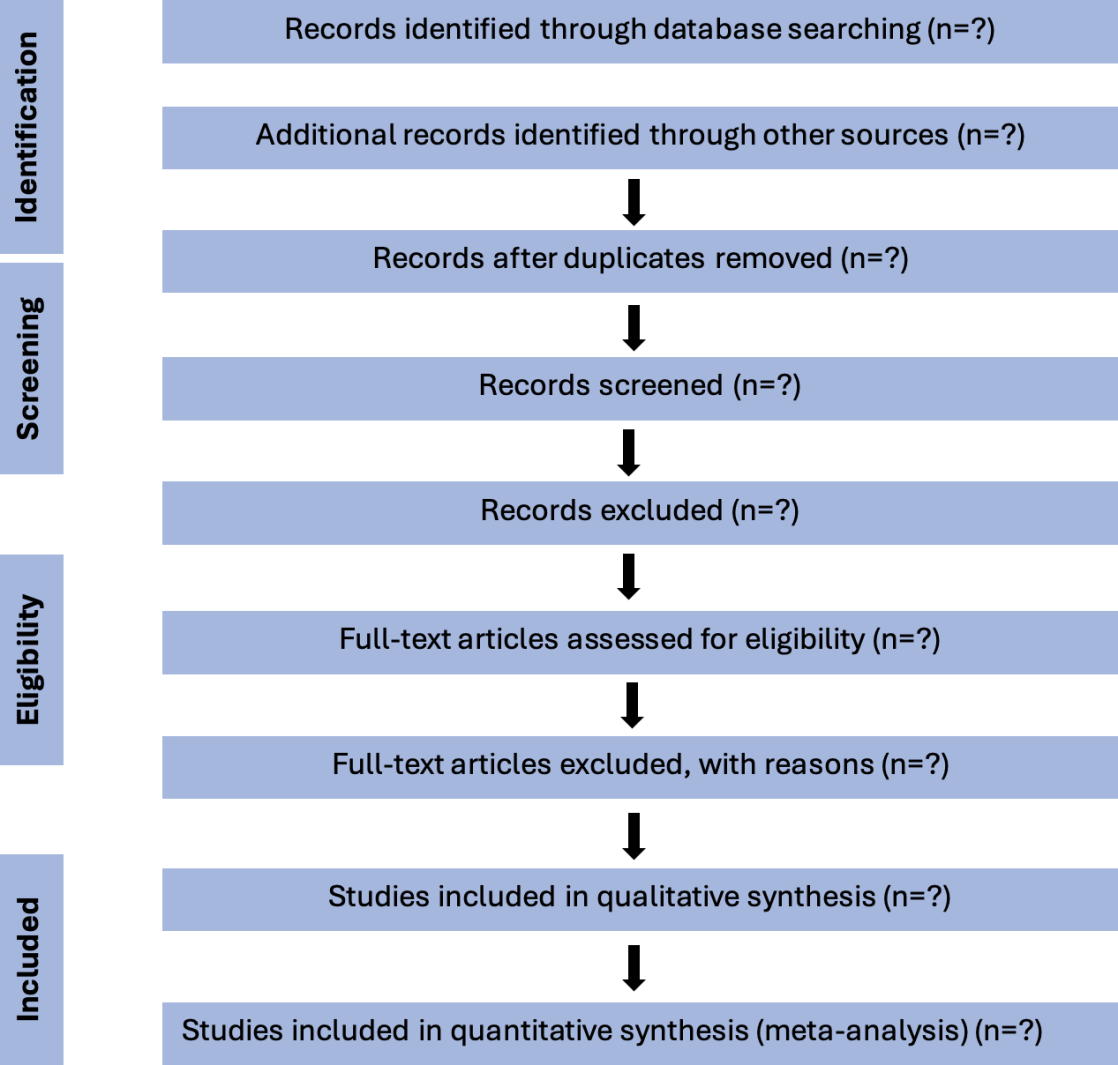
PRISMA flowchart illustrating the systematic search process. PRISMA: Preferred Reporting Items for Systematic Reviews and Meta-Analyses.

The results of the review will be presented using a PRISMA flow diagram, which will show the number of studies identified, screened, excluded, and included (Figure 1). A table displaying key characteristics of included studies will be presented. The quality assessment of the included studies will be tabulated and summarized using the Newcastle-Ottawa Scale. The included studies will be categorized as high, moderate, or low quality. A pooled prevalence estimate will be presented using a forest plot. This will be followed by subgroup analyses, which will explore differences by geographic setting. Predictors of DD will be synthesized either quantitatively or qualitatively. A detailed summary of the key findings and their implications will be reported.

## Discussion

### Principal Findings

The systematic review and meta-analysis will be conducted to determine the pooled prevalence of DD among South Africans with diabetes mellitus. The review will also evaluate the relationship between DD and its determinants. The review will provide an overview of how certain sociodemographic and clinical factors influence the lipid profile in the diabetic population. The findings will be compared with existing data from other African countries and global trends. Notwithstanding the public health burden of DD, there is a lack of systematic reviews reporting on the prevalence of DD in South Africa. This review will reveal significant insight into the public health challenges associated with DD, including its cardiovascular complications. It will assess the various determinants of DD, including sociodemographic factors and health disparities. Providing a comprehensive understanding of these factors will subsequently inform the development of targeted interventions and strategies for the effective management and prevention of DD among the diabetic population, ultimately enhancing health outcomes.

This meta-analysis will yield significant findings that will be used in crafting better tailored inventions for managing DD in South Africa. Given the country’s diverse socioeconomic and cultural landscape, the findings will have the potential to apprise the global strategies for tackling DD. While efforts by the South African health care system to manage complications related to diabetes have shown some advancements, the findings of this review will highlight the need for a more multifaceted approach. This approach will emphasize not only clinical management and treatment guidelines but also public health education, patient support, and community-based programs. In addition, the findings obtained can contribute to the development of future policies, which will offer a beneficial framework for other countries facing similar challenges in mitigating the health burden of DD and its complications.

The implementation of effective policies to manage DD and associated determinants among South Africans with diabetes is significant to the country’s public health strategy. Such strategies could include enhancing screening programs for lipid abnormalities, encouraging adherence to lipid-lowering therapies, incorporating nutritional counseling, and addressing socioeconomic barriers to the effective management of DD. The findings of this systematic review and meta-analysis can inform the development of future policies, thereby enhancing their effectiveness. These can lead to a significant reduction in the prevalence and associated complications of DD, a decrease in health disparities among diabetic populations, and subsequently improve the overall public health outcomes in South Africa. Moreover, the findings may have broader implications for global health strategies, as these successful interventions have been implemented across different populations.

### Strengths and Limitations of This Study

The study protocol outlines a rigorous and transparent methodology for a systematic review and meta-analysis to provide a more detailed understanding of the prevalence and predictors of DD in South Africa. The study protocol offers significant strengths, including adherence to the PRISMA guidelines, a comprehensive and reproducible search strategy, duplicate article screening, and data extraction by independent reviewers (MN and FA), as well as predefined procedures. The study protocol also details quality appraisal tools and statistical methods to improve the reliability of the proposed synthesis. Several limitations of the study protocol should be taken into consideration. First, restricting the search to exclude non-English electronic databases or studies may introduce selection bias, as it excludes relevant published studies in languages other than English. Second, the review is dependent on the methodological quality and completeness of reporting in the available studies, which may limit comparability and synthesis. Third, potential heterogeneity may arise from variations in study designs, populations, and methods for evaluating lipid profiles, which could hypothetically hinder data synthesis and constrain the interpretability of pooled estimates. Finally, the study protocol does not include access to nonindexed regional reports, restricted-access repositories, and private databases, which may influence the comprehensiveness of the evidence base.

## Abbreviations

ACVD: atherosclerotic cardiovascular disease
DD: diabetic dyslipidemia
HDL-C: high-density lipoprotein cholesterol
LDL-C: low-density lipoprotein cholesterol
MeSH: Medical Subject Headings
PRISMA: Preferred Reporting Items for Systematic Reviews and Meta-Analyses
PROSPERO: International Prospective Register of Systematic Reviews
